# Obituary: Claus Nielsen 1938–2024

**DOI:** 10.1186/s12983-024-00528-0

**Published:** 2024-03-12

**Authors:** Jürgen Heinze, Ulrich Technau

**Affiliations:** 1 LS Zoologie/Evolutionsbiologie, Universität Regensburg, Regensburg, Germany; 2 Department for Neurosciences and Developmental Biology, Division of Molecular Evolution and Development, Research Platform Single Cell Regulation of Stem Cells, Faculty of Life Sciences, University of Vienna, Vienna, Austria

Few scholars have left a deep and remaining influence on generations of zoologists. One of them is certainly Claus Nielsen, who recently passed away at the age of 85 years. Claus Nielsen was on the editorial board of Frontiers in Zoology, the journal of the German Zoological Society, for the last 20 years, since 2003. On behalf of the whole Editorial board and the publisher team, we would like to acknowledge the many years of commitment of Claus to our journal.

Claus was born in Copenhagen in 1938, and has for almost all his life studied and worked in Copenhagen. He obtained his Dr. Phil. in 1972 at the University of Copenhagen with a work on entoprocts, for which he became one of the world specialists. After several years of working as a lecturer and Associate Professor at the Zoological Museum in Copenhagen and the University of Copenhagen, he became Professor of Evolutionary Invertebrate Embryology in 2005. At the Zoological Museum, he served as a curator of entoprocts, ectoprocts, phoronids, brachiopods, pterobranchs, enteropneusts as well as later of urochordates and echinoderms.

Claus Nielsen had a world-wide recognition as an expert of marine invertebrates, with a strong interest in ciliary larvae, their evolutionary origin and relationships. Based on his thorough knowledge on all developmental forms of marine invertebrates, he was convinced that the common ancestor of Bilateria evolved from such ciliary larvae. His book “Animal Evolution: Interrelationships of the Living Phyla” published by Oxford University Press in 1995 was a guide and standard textbook for many evolutionary biologists and zoologists for several decades and remains influential up to this day. Claus received many honors, among them the prestigious Alexander Kovalevsky medal of the St. Petersburg Society of Naturalists (2001). In 2006 he became a foreign member of the Linnean Society of London and received the Linnean Medal for Zoology in 2015. He was an honorary member of the Society for Integrative and Comparative Biology and of the International Society for Invertebrate Morphology. He served in many academic committees and review panels. What is more, he was a fantastic, inspiring teacher in numerous field courses at marine stations, where his enthusiasm and witty charm has inspired and motivated generations of students. I also witnessed his tireless fascination and love for marine organisms, as well as his humor in practical courses taught to international Ph.D. students at the Marine station in Kristineberg, Sweden. His spirit, his scholarship and his friendly nature will be missed.

